# Serum 25-Hydroxyvitamin D and Diet Mediates Vaso-Occlusive Related Hospitalizations in Sickle-Cell Disease Patients

**DOI:** 10.3390/nu10101384

**Published:** 2018-09-29

**Authors:** Michael L. McCaskill, Olalekan Ogunsakin, Tete Hottor, Emily W. Harville, Rebecca Kruse-Jarres

**Affiliations:** 1 Department of Global Environmental Health Sciences, Tulane University School of Public Health and Tropical Medicine, New Orleans, LA 70112, USA; Olalekan.Ogunsakin@uthct.edu (O.O.); henkht@yahoo.com (T.H.); 2 Department of Epidemiology, Tulane University School of Public Health and Tropical Medicine, New Orleans, LA 70112, USA; eharvill@tulane.edu; 3 Section of Hematology and Medical Oncology, Tulane University School of Medicine, New Orleans, LA 70112, USA; rebeccakr@bloodworksnw.org; 4 University of Washington, Washington Center for Bleeding Disorders at Bloodworks Northwest, Seattle, WA 98195, USA

**Keywords:** sickle cell disease, sickle cell crisis, vitamin D, diet

## Abstract

Sickle cell disease (SCD) is a genetic disorder resulting from the presence of mutated hemoglobin S (HbS). Homozygous carriers will present with early manifestations of painful vaso-occlusive crises. SCD patients have been reported to be severely deficient in vitamin D (<20 ng/mL). Four years (2010–2014) of individual de-identified Sickle Cell Clinic of Southern Louisiana (SCCSL-SCD) patient records were analyzed for vitamin D status and the level of crisis-related ER/hospital utilization. To determine the dietary, and behavioral mediators of SCD-crisis in our study population, a cohort of 102 SCCSL-SCD patients were administered a survey that evaluated sun exposure, dietary behaviors, and pain frequency and severity. Patients with circulating levels of 25(OH)D_3_ less than 14.1 ng/mL reported having more crisis-related hospital visits per year (10) than patients with 25(OH)D_3_ serum levels >34 ng/mL. The result of the dietary survey detailed a relationship between patients who reported to have “Almost Never” consumed fish or milk in their diets and more frequent hospital stays and ER visits than those who reported consuming these products on a daily basis. Those who consumed these foods in their diet several times a month also had fewer ER visits when compared to the “Almost Never” category.

## 1. Introduction

Sickle cell disease (SCD) is a genetic disorder that is caused by the improper insertion of the amino acid valine in place of glutamate at position 6 of the hemoglobin chain [1,2,3]. This seemingly small error results in significant hemoglobin instability, solubility changes, and morphological changes in the form of misshapen red blood cells that are incapable of normal oxygen exchange. SCD presents most frequently among people of black African descent; however, the heterozygous form is a genetic adaptation to increase malaria resistance, and it can be found anywhere that malaria is endemic, e.g., Africa, the Middle East, the eastern Mediterranean region, and India [2,4]. The heterozygous HbS gene is present among approximately 8% of African-Americans; 1 out of 500 births will have homozygous HbSS/SCD. One out of 36,000 Hispanic child births will have HbSS/SCD, making it the most common inherited blood disorder in the United States. Although the heterozygous HbS carrier is asymptomatic and benefits from resistance to the parasite that causes malaria, the much rarer homozygous HbSS carrier will present with early manifestations of vaso-occlusive crises, which are characterized by generalized body, bone, and joint pains [3,5].

Vitamin D is a fat-soluble vitamin naturally present in very few foods, added to others, and available as a dietary supplement. Because most humans can receive adequate levels of vitamin D from sun exposure, vitamin D is often considered to be a prohormone as opposed to a true vitamin [6]. Although there is insufficient evidence to prove that vitamin D supplementation will prevent bone fractures in healthy vitamin D sufficient populations, in vitamin D insufficient populations, vitamin D is important in bone mineralization, and deficiency can result in bone fractures, musculoskeletal pains and muscle weakness [7,8,9]. Recent studies have shown marked vitamin D (25(OH)D_3_) deficiency among SCD patients [4,10,11]. Rovner et al. (2008) found that African American children with SCD had higher rates of vitamin D deficiency when compared to non-SCD African-American children living in the same neighborhoods, and this same report suggested that poor diet that is related to low socioeconomic status was not associated with observed vitamin D deficiency. Several mechanisms have been suggested as the likely cause of the low serum 25(OH)D_3_ concentrations among SCD patients, such as low cutaneous synthesis, decreased intestinal absorption, disturbance of adipose tissue metabolism, and chronic hemolysis, resulting in bilirubin deposition, among others [1,2,3]. Variability in SCD severity may also be driven by the presence of comorbidities that are frequently underdiagnosed and can influence symptom severity.

**Figure 1:**
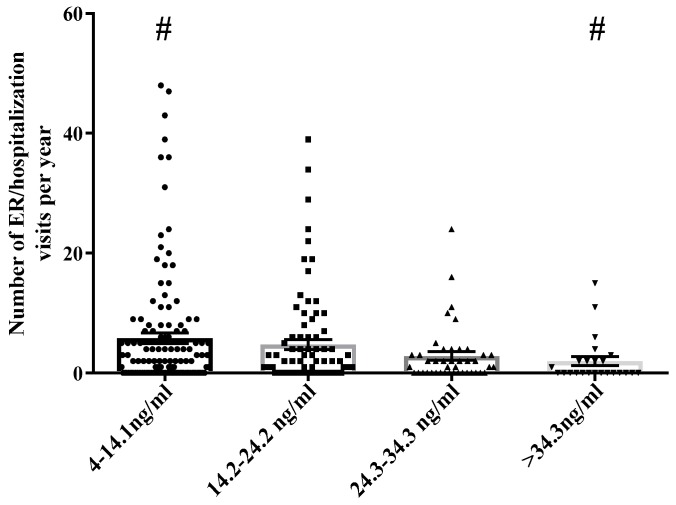
Serum 25(OH)D_3_ levels and sickle cell crisis related hospital visits in SCD patients. In an evaluation of ~1500 sickle cell disease (SCD) patient medical records over a 4-year period it was observed that SCD patients with severe vitamin D deficiency (<14.1 ng/mL) had on average more than 10 hospital visits/year. SCD patients with documented higher levels of circulating vitamin D (>34 ng/mL) had statistically significant less than two hospital visits/year. # *p* < 0.05 one-way ANOVA compared to the lowest circulating vitamin D group.

There are also dietary mediators of SCD severity [12]. People who suffer from SCD have nutrient availability complications due to hyper-reactive hematopoietic activity and rapid nutrient turnover [13,14]. Hasanto et al. found significant deficiencies among SCD patients in vitamins A, C, and E as well as zinc, and elevated serum copper. These deficiencies in essential nutrients increase systemic oxidative stress, which may contribute to the severity of sickle cell manifestations. In particular, vitamin D is an important mediator of sickle cell crisis severity. Rovner et al. (2008) found that SCD children have five times the risk of vitamin D deficiency as compared to healthy children, vascular inflammation [10], and the authors have observed low serum vitamin D levels to be associated with increased hospitalization rates (Figure 1).

Vitamin D can be acquired in two different ways. The D_2_ form of vitamin D is called ergocalciferol, and it is typically found in non-animal sources, such as mushrooms, plants, and some supplements. Vitamin D_3_ or cholecalciferol is found in animal sources, such as fatty fish, egg yolks, liver, or also from supplements. In humans, cholecalciferol acquisition is primarily managed through exposure to the ultraviolet B rays in sunlight. Both forms of vitamin D are then converted to 25(OH)D_3_ in the liver, which is further converted in the kidneys to 1,25(OH)_2_D_3_ by 1-alpha-hydroxylase. This active form of vitamin D, 1,25(OH)_2_D_3_, promotes calcium absorption in the small intestine, increases bone mineralization through calcium mobilization, and decreases parathyroid hormone (PTH) secretion by the parathyroid glands [15]. Vitamin D status is typically measured by 25(OH)D_3_ levels in which bone disease is associated with levels below 10 ng/mL. Optimal levels are higher than 20 ng/mL, and suppression of PTH is sometimes observed at levels >30 ng/mL [15].

A comparison study of data from the National Health and Nutrition Examination Survey (NHANES) III performed in 1988–1994 to data from NHANES 2001–2004 showed an overall increase from 2% to 6% in vitamin D deficiency-designated as 25(OH)D_3_ levels less than 10 ng/mL—in populations of non-Hispanic Whites, Mexican-Americans, and non-Hispanic Blacks. Non-Hispanic Blacks further showed an increase from 9% to 29% prevalence of 25(OH)D_3_ deficiency and continued to have the largest number of subjects with 25(OH)D_3_ deficiency [16]. Furthermore, in 50% of the non-Hispanic Black study participants in the NHANES data, vitamin D levels were <17.3 ng/mL compared to 86% of the patients surveyed by Goodman et al. [1].

Data presented in this manuscript detail the association of environmental exposures, diet, vitamin D status, and crisis events by assessing the relationship between nutritional status and hospitalization rates in SCD patients. Although the primary *a priori* hypothesis for the analysis of this data was to determine if serum levels of vitamin D are inversely related to vaso-occlusive related hospitalization rates in sickle cell anemic patients of the Sickle Cell Clinic of Southern Louisiana, the overall goal of this study was to identify environmental mediators of severe vaso-occlusive crisis. With this knowledge, possible novel therapies may be developed that might lead to extended remission periods, reduced infection morbidity, and less severe SCD crises.

## 2. Materials and Methods

This manuscript presents two studies designed to determine the relationship between serum vitamin D, diet and vaso-occlusive crises-related hospitalizations in the SCCSL patient community. The first study was designed to establish via a retrospective longitudinal medical record analysis, a baseline understanding of the relationship, direction, and magnitude of the correlation between vitamin D levels, and crisis-related hospitalization rates in SCCSL patient community. The second study was a survey administered to a smaller subset of the SCCSL patient population designed to elucidate further the relationship between vitamin D, hospitalization rates, vitamin D-modulating diet, supplementation, and vitamin D regulating behaviors, such as time spent outdoors and outside activity.

1. We defined the Tulane Retrospective Longitudinal Sickle Cell Disease Study group (TRLSCD), as patients treated at the SCCSL (Sickle Cell Center South Louisiana) who had at least one crisis event in the previous four years (2010–2014), as defined as in the medical records and International Classification of Disease ninth revision as “sickle cell with crisis or with vaso crisis” (ICD9 CM 282.62/64) and at least one recorded blood serum vitamin D report per year (*n* = 1531), as recorded in medical records. The defined deidentified TRLSCD sample group was divided into quartiles by mean levels of serum 25(OH)D_3_. Serum 25(OH)D_3_ values 2.5 times the standard deviation were excluded from the statistical analysis (*n* = 2). Quartiled SCD groups delineated by 25(OH)D_3_ levels allowed us to elucidate the relationship between low serum 25(OH)D_3_ levels and the frequency and cumulative length of infirmed time (Emergency Department and hospital visits over 12 months).
2. Between 2009 and 2010, during scheduled healthy check-ups at the Sickle Cell Clinic of Southern Louisiana, and inpatient units at the Tulane Medical Center, 102 sickle cell disease patients, were recruited to participate in a food/supplement frequency, pain and outdoor activity questionnaire. Of the original cohort of recruited patients, one patient withdrew from the study and another declined to give consent resulting in 100 sickle disease patients who agreed to participate in the study, provided informed consent, and completed the study. Surveys were administered by a health care provider in a clinical setting to study participants according to the IRB approved protocol.

The Tulane University Institutional Review Board approved the protocols of both the retrospective longitudinal study protocol and the dietary survey. All of the participants provided written informed consent. The inclusion criteria for the retrospective longitudinal study and dietary and behavioral study were age (at least 18 years at year 1 of the 4 year retrospective study), confirmed diagnosis of sickle cell disease (regardless of subtype-SS, SC, S/thalassemia), and at least one medically documented 25(OH)D_3_ status per year. SPSS version 24 [17] was utilized to analysis statistical relationships in both the survey study and medical record review. ANOVA was used to determine the statistical significance of hospital stay lengths per 12 months, as a function of vitamin D status.

### Surveys

Three surveys were administered to patients evaluating pain levels, diet, and outdoor activity levels. The pain level survey inquired about pain characteristics pre and post vitamin D replacement therapy. Specifically, questions asked about mild to moderate pain frequency and severity rating, vaso-occlusive pain episodes frequency and severity rating, hospitalization frequency for sickle cell-related pain, pain medication intake frequency, and whether or not vitamin D replacement seemed to make any difference.

The diet survey asked about the intake frequency of foods high in vitamin D, particularly fish, milk, cheese, and eggs.

Patients were asked four dietary questions adapted from NHANES dietary questionnaire tools. i.e., “How often do you eat fatty fish (such as catfish, salmon, eel, mackerel, tuna, sardines)?”, “How often do you drink at least one glass of milk?”, “How often do you eat cheese (at least one slice of Kraft singles or the equivalent)?”, “How often do you eat eggs?” Food frequency answer options were coded as [1] “daily (at least one meal a day)”, [2] “several times per week” [3], “several times per month” [4], “several times per year”, and [5] “almost never”.

Survey questions inquired about how often vitamin D supplements are taken. The outdoor activity questionnaire asked about how many days in a month that patients stay in bed at home, how much time they spend outdoors on a daily basis, if they walk or exercise regularly outdoors, if they work outdoors, and if they go to school.

Correlation coefficients were used to examine the relationship between indicators. To minimize recall bias, post vitamin D supplementation survey data was utilized in this analysis when applicable.

## 3. Results

Vitamin D serum levels in the surveyed population were inversely associated with medical record reported ER/Hospitalization visits (*r* = 0.175, *p* = 0.04) (Table 1). Self-reported *How Often Hospitalized* responses were positively associated with serum vitamin D levels (Table 1) (*r* = 0.224, *p* = 0.01). Correlation analysis of survey data (Table 1) also indicated an inverse relationship between *Time Spent Outside* and *How Often Hospitalized* (*r* = 0.187, *p* = 0.04); as SCD patients spent more time outside they reported to spend fewer days in the hospital. This data also indicates that *Working or Exercising Regularly* had no statistically relevant association with self-reported hospitalizations (*r* = 0.007, *p* = 0.48). There was also no significant difference in the mean self-reported hospitalization rates between those answering “yes” or “no” to being prescribed vitamin D (Table 1).

Responding “yes” to vitamin D treatment was not significantly associated with sufficient serum vitamin D levels (>20 ng/mL). There was no significant association between the self-reported prescription of vitamin D supplements and having serum vitamin D levels >20 ng/mL (22% sufficient vs. 13% sufficient, *p* = 0.334) (Table 2).

In an evaluation of ~1500 SCD medical records, it was observed that >90% of the TRLSCD study group had serum levels less than 34.3 ng/mL. TRLSCD patients with severe vitamin D deficiency (<14.1 ng/mL) in the lowest quartile had on average more than 10 hospital visits/year compared to SCD patients in the highest quartile of serum vitamin D levels. SCD patients with documented higher levels of serum vitamin D (>34.3 ng/mL) had statistically significantly less than two hospital visits/year compared to the lowest serum vitamin D quartile.

**Figure 2:**
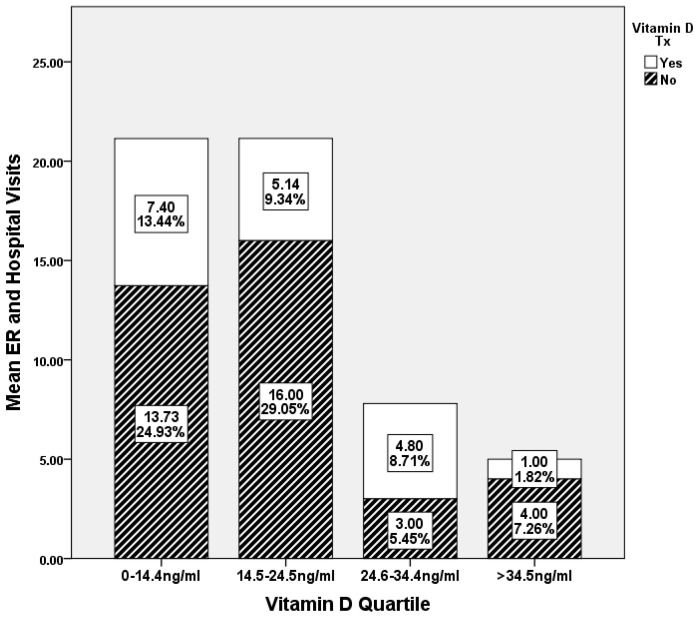
ER/Hospital Visits in SCD Patients Juxtaposed Against Vitamin D Quartile and Treatment Status. Supplemented cholecalciferol levels were categorized by quartiles of circulating vitamin D levels in surveyed sickle cell disease patients (*n* = 102). In the Tulane Retrospective Longitudinal Sickle Cell Disease Study group (TRLSCD) group medical records of over 4 years of Sickle Cell Disease patients, the absence of vitamin D treatment in patients is correlated with the serum levels >34 ng/mL. In addition, vitamin D treatment (≥1000 IU daily) corresponds with ~50 to 70% less mean levels of SCD-related crisis per year in patients with circulating vitamin D levels lower than 24 ng/mL.

When comparing the emergency department (ED) and hospital visits of TRLSCD patients to vitamin D quartile and treatment status, 76.8% of the surveyed population was under 24.3 ng/mL of serum vitamin D levels. Sixty-six percent of SCD patients in the lowest quartile of serum vitamin D were not prescribed nor were they independently taking vitamin D supplements. Study participants in the lowest quartile of serum vitamin D levels (0–14.4 ng/mL) and not prescribed vitamin D supplements had nearly twice as many hospitalizations (13.73 ED visits) in a twelve-month period as compared to the patients that were prescribed vitamin D within the same quartile (7.40 ED visits). In the second serum vitamin D quartile (14.5–24.5 ng/mL) patients not prescribed vitamin D had over three times the hospitalizations (16 ED visits) as compared to SCD patients in the quartile prescribed vitamin D (5.14 ED visits). In the third serum, vitamin D quartile (24.6–34.4 ng/mL) SCD patients prescribed vitamin D recorded a slightly higher increase in hospitalizations (4.8 ED visits) per twelve- month period as compared to patients not prescribed vitamin D (three ED visits). However in the highest serum vitamin D quartile (>34.5 ng/mL) SCD patients not prescribed vitamin D had four times as many hospitalizations (four ED visits) as compared to SCD patients that were prescribed vitamin D (one ED visit) (Figure 2).

**Figure 3:**
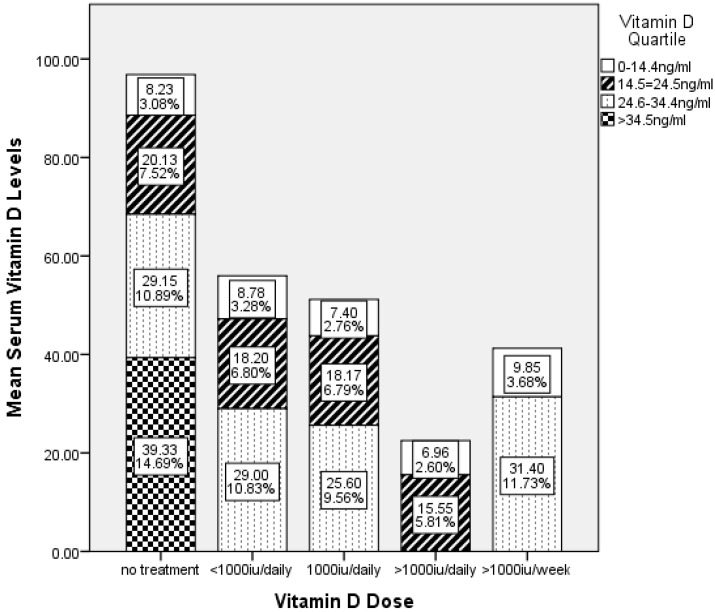
Mean Serum Vitamin D Levels in the Surveyed SCD Patients Compared Vitamin D Quartile and Treatment Dosage Sixty-four percent of the 102 patients surveyed were prescribed cholecalciferol at least daily or weekly. Fifteen percent of the study participants were in the highest quartile and were not treated with cholecalciferol.

When comparing serum vitamin D levels to prescribed vitamin D treatment dose, 36% (*n* = 37) of the 102 surveyed SCD patients were not prescribed vitamin D supplements, and 40% percent of the non-vitamin D prescribed group of surveyed SCD patients had serum vitamin D levels >34 ng/mL (Figure 3). Of the SCD patients that were prescribed vitamin D supplements by the medical providers, 52% of the patients prescribed <1000 IU/day, 50% of the patients prescribed 1000 IU/day, and 76% of the patients prescribed >1000 IU/week fell into the 3rd quartile (24.6–34.4 ng/mL) of serum vitamin D levels. One hundred percent of the patients prescribed >1000 IU/day were in the bottom two quartiles (<24.6 ng/mL). Levels of serum vitamin D greater than 14.1 ng/mL were not statistically associated with being prescribed vitamin D supplements at any of the dosages reported. In fact, 66.7% of the patients who were prescribed vitamin D supplements had vitamin D serum levels of less than 14.1 ng/mL and they were in the lowest quartile of surveyed patients.

**Figure 4:**
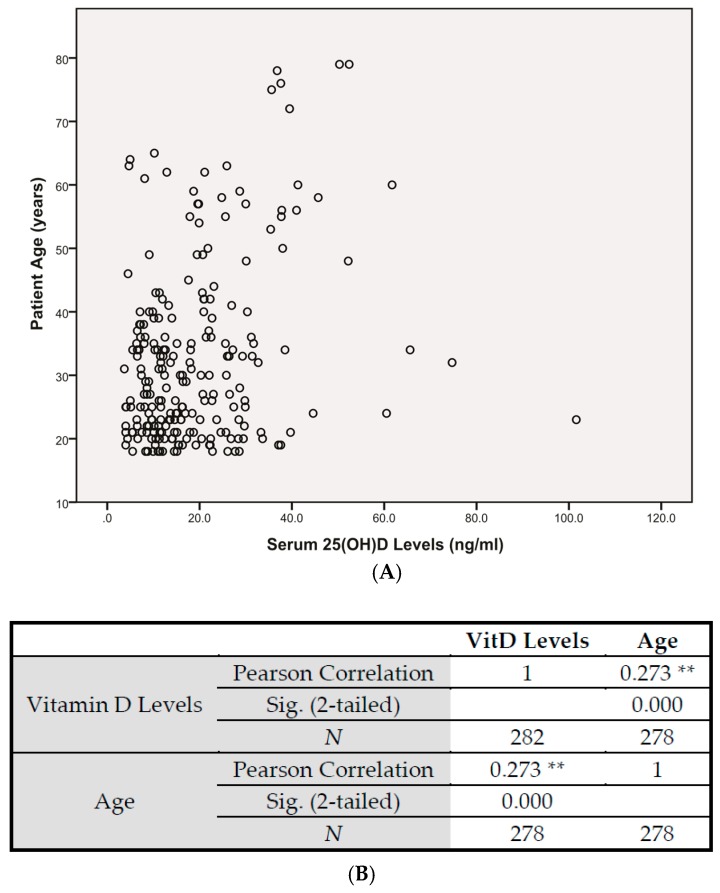
Histogram and Correlation Analysis of Serum Vitamin D Levels and Sickle Cell Patient Age In the TRLSCD study group, medical records from over 1500 sickle cell southern Louisiana patients were reviewed for statistical significant correlation of age to serum vitamin D. (**A**) Seventy-five percent of the patients reviewed were under the age of 35. (**B**) There is no significant association between age and vitamin D levels. ** Pearson Correlation is significant at the 0.01 level (2-tailed).

Medical records from over 1500 sickle cell southern Louisiana patients were reviewed for an association between age and serum vitamin D levels. Seventy-five percent of the patients reviewed were under the age of 35. With the left skew of age distribution being observed, there is no significant association between age and vitamin D levels (Figure 4).

A Levene’s Test for Equality of Variances and *t*-test for Equality of Means was conducted to determine the level of association between gender and serum vitamin D levels in the TRLSCD study group. Gender has no effect on vitamin D status in the studied population, and nearly 97% of the TRLSCD study population self-identifies as Black or African American. No statistical significance in the serum vitamin D levels was observed between the self-identified racial groups.

**Figure 5:**
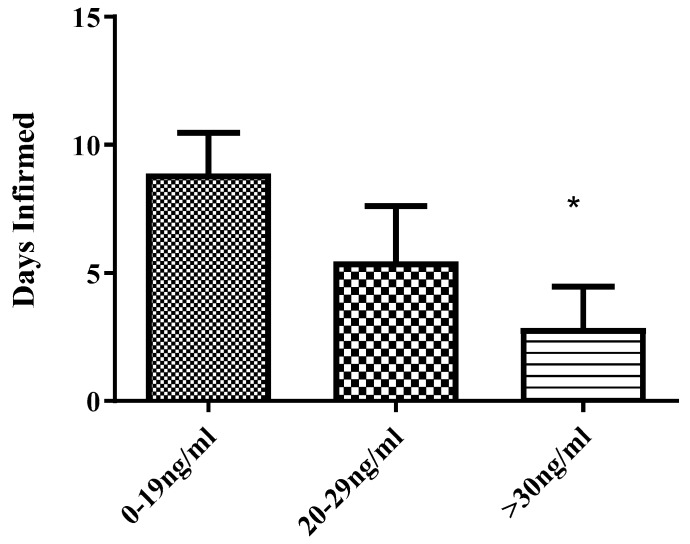
Hospitalizations and circulating 25(OH)D_3_ levels in surveyed sickle cell disease-Sickle Cell Clinic of Southern Louisiana (SCD/SCCLS) patients. Serum vitamin D 25(OH)D_3_ was quantified from the blood serum of 102 SCA patients. Mean hospitalizations over a 12 month period were used to generate days infirmed data. Those with severe deficiency (0–19 ng/mL/mg) had most days infirmed (nine days), mild to moderat deficiency (20–29) ng/mL/mg) had an average of 5 days infirmed and “not deficient” group were less than three days infirmed. Student’s *t*-test was used to generate significance, * *p* < 0.05 compared to lowest circulating vitamin D group.

The SCCSL/SCD survey results had shown a statistically significant reduction of nearly seven days per month in ED/hospital utilization when vitamin D/25(OH)D_3_ levels were over 30 ng/mL (Figure 5). Serum vitamin D 25(OH)D_3_ was quantified from the blood serum of 102 SCD patients. Mean hospitalizations over a 12-month period were used to generate days infirmed data. Those with severe deficiency (0–19 ng/mL/mg) had most days infirmed (nine days), mild to moderate deficiency (20–29 ng/mL/mg) had an average of five days infirmed and “not deficient” group were less than three days infirmed.

**Figure 6:**
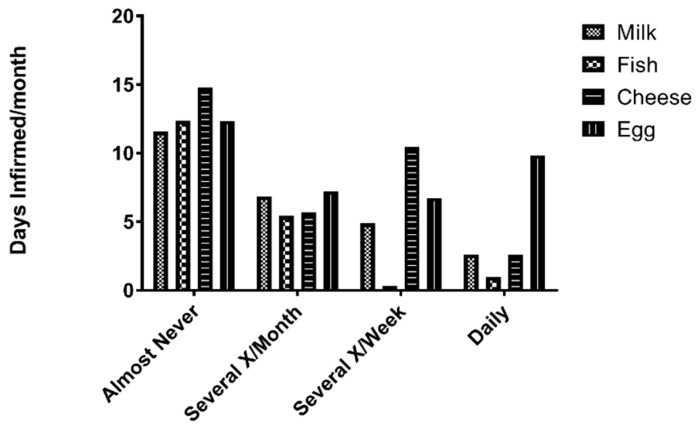
Fish, Milk, Egg, and Cheese Consumption vs. Crisis-related Hospitalizations. Self-reported dietary consumption data, along with vitamin D and hospitalization rates were quantified from 102 sickle cell anemic SCCLS patients to quantify the consumption of foods commonly associated with dietary vitamin D homeostasis. When survey results were compared with serum 25(OH)D_3_ levels, 12 months of mean aggregate hospital utilization data and diets high in fish, milk, cheese, and eggs was associated with a lower number days of being hospitalized.

Utilizing 12 months of self-reported dietary survey data of 102 patients as compared to self-reported SCD patient days infirmed, patients who answered “Almost Never” to milk or fish consumption had on average 12 (milk) and 14 (fish) days infirmed per month. Patients who answered “Several x/Month” to milk or fish had on average seven (milk) and eight (fish) days infirmed per month. Patients who answered “Several x/Week” had on average five (milk) and one (fish) days infirmed per month. Patients who answered “Daily” to consumption of milk or fish had on average three (milk) and less than one (fish) days infirmed per month (Figure 6).

Patients who answered “Almost Never” to eggs or cheese consumption had on average 13 (eggs) and 15 (cheese) days infirmed per month. Patients who answered “Several x/Month” to eggs or cheese had on average six (eggs) and eight (cheese) days infirmed per month. Patients who answered “Several x/Week to the consumption of eggs or cheese had on average six (eggs) and 10 (cheese) days infirmed per month. Patients who answered “Daily” to consumption of eggs and cheese had on average three (eggs) and nine (cheese) days per month (Figure 6).

**Figure 7:**
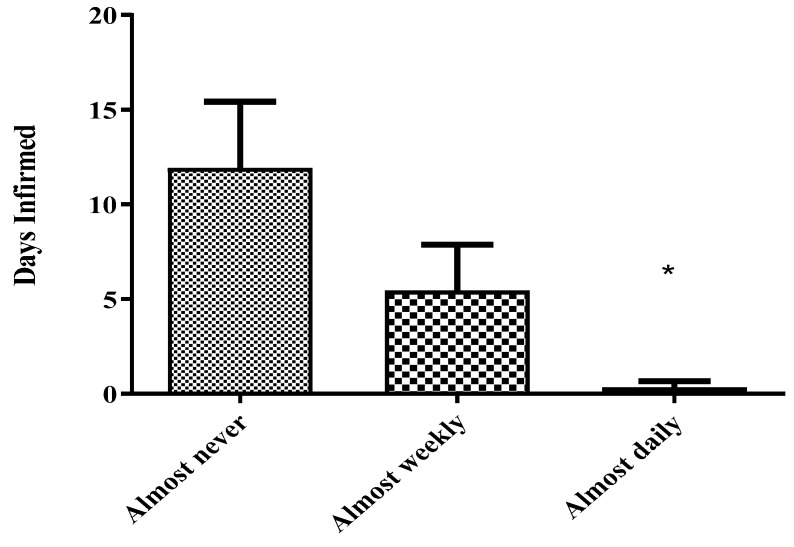
Hospital utilization and fish consumption in severely vitamin D deficient sickle anemic populations. Self-reported dietary consumption data, along with vitamin D and hospitalization rates were quantified from 102 sickle cell anemic SCCLS patients to quantify the consumption of fish. When survey results were compared with serum 25(OH)D_3_ levels, 12 months of mean aggregate hospital utilization data and diets high in fish, was associated with lower total lengths of hospitalization, even in vitamin D deficient populations. Student’s *T*-test was used to generate significance. * *p* < 0.05 as compared to lowest circulating vitamin D group.

Sickle cell disease patients in the lowest tertile of serum vitamin D who self-reported to consume fish daily ultimately had statistically significant fewer days infirmed (<1) as compared to vitamin D deficient SCD patients who self-reported to “Almost Never” consume fish (>12) (Figure 7).

## 4. Discussion

**Figure 8:**
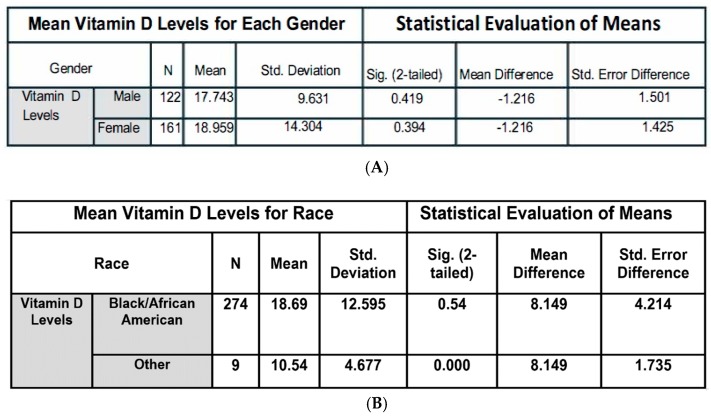
Gender and Race Serum Vitamin D Correlation Analysis Medical records from over 1500 sickle cell southern Louisiana patients were reviewed for serum vitamin D levels. Levene’s Test for Equality of Variances and *t*-test for Equality of Means was conducted to determine the level of association between gender and serum vitamin D levels in the TRLSCD study group. (**A**) Gender status had no statistical effect on serum vitamin D levels. (**B**) Approximately 97% of the TRLSCD study population self-identify as Black or African American. No significant significance was observed between racial groups.

This two-part study was conducted to determine the nature of the relationship between dietary behaviors of SCD patients and painful crisis resulting in hospitalizations. An inverse association between length of hospital utilization and consumption of a healthy diet rich in vitamin D has been demonstrated in several studies [15,18,19]. The majority of people who suffer from SCD are chronically and severely deficient in vitamin D (<20 ng/mL) [1]. Respiratory infections are one of the most common illnesses in North America [20], and infection has been reported to be the single most common initiator of an SCD crisis event [12]. Vitamin D has been shown to lower systemic inflammation, as measured by IL-6 and TNF-α via the MPK-1 pathway [21,22]. Also, Zhang Yong et al. found that levels of 30 and 50ng/mL of 25(OH)D_3_ significantly inhibited inflammatory mediators IL6 and TNF-α, while levels that were lower than 20 ng/mL had no detectable anti-inflammatory activities [23]. In a racially homogenous population with non-statistical differences in gender (Figure 8), the retrospective review of four years of medical records determined that SCD crisis-related hospitalizations/ED visits and vitamin D levels are inversely related. This observation was substantiated by the statistically significant stepwise reduction in SCD-related crises as vitamin D levels increased through the top three quartiles observed in the survey population. Of the studied SCCSL/SCD population, >90% had serum levels of vitamin D lower than 34 ng/mL (Figure 1), which is lower than the medically recommended serum vitamin D levels [24]. The dietary, pain, and behavior survey also revealed that SCD patients with 25(OH)D_3_ levels that were less than 17 ng/mL have on average more costly hospitalizations, and this increase of seven days infirmed per month has a quantifiable effect on quality of life as measured by other covariates surveyed in this study such as time spent outdoors. The authors believe that it is possible many SCD crisis events are triggered by respiratory infections, which have been shown in the literature to be attenuated by immune system modulating levels of circulating vitamin D. Reductions in time spent outdoors might inversely affect vitamin conversion and ultimately the patient’s immune response to infection.

As our survey data suggests, diets that are high in fish, milk, cheese, and eggs were associated with reductions in hospital/ER visits when compared to those who consume less or none of these foods in their diets (Figure 6). Even SCD patients in the lowest tertile of circulating vitamin D who self-reported to consume fish daily ultimately had statistically significant fewer days infirmed (<1) as compared to vitamin D deficient SCD patients who self-reported to “Almost Never” consume fish (>12) (Figure 7). Diets that were high in fish were associated with shorter hospital stays, a confirmation that was documented in other similar studies [16,25,26]. The authors believe vitamin D’s role in improving the health of SCD patients may involve the modulation of immune response to respiratory infections along with the reduction of the presence of systemic reactive oxygen species in the patient. Vitamin D-mediated systemic inflammatory modulation has been reported in similar studies [27,28]. Irrespective of mechanism, these studies indicate that not only does diet have a quantifiable role in modulating SCD crises but circulating levels of vitamin D also have an inverse relationship with hospital/ED utilization in SCD patients. Moreover, based on the dietary data, frequent fish consumption also plays a role in reducing the number of hospitalizations per year in this crisis vulnerable population (Figure 7).

### Cholecalciferol/Vitamin D Treatment

These studies reveal two noteworthy aspects concerning the relationship between vitamin D and SCD-related hospitalizations. First, according to survey and medical report findings, to achieve lower SCD crisis-related hospitalization rates serum vitamin D levels must be maintained in a sufficient range. Although there is no medically definitive overall optimal serum vitamin D level, recent publications have presented optimal serum vitamin D levels for maximum calcium absorption to be greater than 34 ng/mL [29] and serum levels of vitamin D that are lower than 20 ng/mL have been clinically observed to suppress parathyroid hormone synthesis [30]. However, as a general health reference, the Mayo Medical Laboratories reference ranges for total serum 25-hydroxyvitamin D defines serum vitamin D levels below 25 ng/mL to be deficient with optimal serum vitamin D ranging from 25 to 80 ng/mL [25]. Our data support these assertions, as measured by statistically lower SCD crisis-related hospitalization rates in serum vitamin D groups with vitamin D levels that were greater than 24 ng/mL.

Second, vitamin D supplementation was not statistically associated with reduced hospitalizations, and hospitalization rates were statistically inversely related to serum vitamin D levels in the surveyed population. Cholecalciferol supplementation greater than 1000 IU/day, although not statistically significantly associated with reduced hospitalizations (Table 1), or significantly associated with serum vitamin D levels above 20 ng/mL (Table 2), did result in a higher percentage of SCD patients achieving levels of serum vitamin D levels greater than 24 ng/mL. Due to the lack of a statistically significant association between vitamin D supplementation and sufficient vitamin D status (>20 ng/mL) the prescribed daily vitamin D supplementations of 1000 to 2000 IU are likely not adequate to increase serum vitamin D levels of “vitamin D insufficient” SCD patients to reclassify them into being “vitamin D sufficient” (Table 2 and Figure 2). This being stated, the surveyed SCD patients in the lowest two quartiles of serum vitamin D who answered “yes” to receiving and participating vitamin D supplementation had on average 50% and 75% less SCD-related hospitalizations, respectively, when compared to patients in the same quartile who answered “no” to vitamin D supplementation. Also, it is important to note that patients with no medical record of vitamin D treatment had on average three times higher hospitalization rates in a 12-month period as compared to vitamin D receiving patients within the same quartile. Although hospitalization rates in non-vitamin D prescribed groups were on average three times higher as compared to medically treated SCD patients, hospitalization rates reduced significantly as serum vitamin D quartiles increased irrespective of vitamin D treatment status. The authors believe this lack of observed substantive increase in serum vitamin D levels in vitamin D supplemented patients may be associated with the characteristically high metabolic state of SCD patients. It is plausible that as the systemic need for vitamin D reaches a critical state, supplemented cholecalciferol is utilized and metabolized quickly after treatment resulting in beneficial effects, but leaving very little vitamin D binding protein sequestered 25(OH)D_3_ to be quantified clinically. Also, with the lack of individual records of serum vitamin D levels prior to vitamin D supplementation, temporal individual vitamin D flux over time and within one quartile is undetermined. As seen in Figure 2, with nearly 50% and 68% fewer days infirmed for vitamin D treated 1st and 2nd quartile SCD patients, respectively, the data support that vitamin D treatment of critically low SCD patients could result in quantifiable improvements in a health outcomes, such as hospitalization rates, without corresponding significant increases in circulating vitamin D.

These data suggest that more frequent and/or increased doses of vitamin D maybe be necessary to quantifiably increase serum vitamin D levels into a “sufficient” state. This being stated the authors recognize that vitamin D measurements are a snapshot clinical observation of a biometric in constant flux. It is possible that patients prescribed vitamin D may have been originally among the patients with the lowest clinical serum vitamin D levels, prompting their medical provider to prescribe and encourage supplementation.

The limitations of this study were small dietary and behavioral survey size. Self-reporting and recall bias are also limitations of this study. There were covariate correlations that were impaired by the low number of survey responses. The authors also recognize that vitamin D measurements are a snapshot clinical observation of a biometric in constant flux. Only the post vitamin D treatment survey responses were utilized for analysis in this study as the authors recognize this study is influenced by recall bias and the lack of data concerning prior illness severity.

## 5. Conclusions

Data ascertained from this subset of SCD patients offers promising opportunities for future research into diet, and vitamin D mediated SCD crisis reduction. Although the data appears to confer that even the most severely vitamin D deficient population can improve ED/hospital utilization rates via dietary modifications, the authors acknowledge that there are unmeasured confounders that could be contributing to this dietary association with decreased ER visits, including behaviors that are connected to high levels of fish consumption, which could be working independently to improve outcomes. The authors also acknowledge that there is an amalgam of behaviors, genetic expression, and environmental pressures that fully influence human health, and it is appropriate to consider that there are possibly other unmeasured lifestyle and behavioral mediators in SCD patients, which may be influencing our observations. This being stated, this study’s observations further substantiate the concept that SCD crises can be modulated by diet and reinforce the importance of maintaining a healthy vitamin D levels for SCD patients, even among severely vitamin D deficient subjects.

## Figures and Tables

**Table 1:** Sickle Cell Patient Survey Questionaire and Medical Record Data Correlation Table.

		Fish Diet	Milk Diet	Cheese Diet	Serum Vitamin D Levels	ER and Hospital Visits	Vitamin D Tx	Hospitalization Rates	VitD Dose Freq.	Vitamin D Dose per Week
Fish Consumption	Person Correlation	1	0.028	0.132	−0.156	0.089	−0.076	0.101	0.080	−0.044
	Sig. (1-tailed)		0.397	0.107	0.071	0.202	0.246	0.172	0.274	0.339
	*N*	90	90	90	90	90	84	90	59	90
Milk Consumption	Person Correlation	0.028	1	0.399 **	−0.251 **	0.274 **	0.096	0.028	0.055	0.073
	Sig. (1-tailed)	0.397		0.000	0.008	0.004	0.193	0.396	0.340	0.248
	*N*	90	90	90	90	90	84	90	59	90
Cheese Consumption	Person Correlation	0.132	0.399 **	1	−0.082	0.140	−0.078	0.095	−0.009	−0.025
	Sig.(1-tailed)	0.107	0.000		0.220	0.093	0.239	0.186	0.472	0.408
	*N*	90	90	90	90	90	84	90	59	90
Serum Vitamin D Levels	Person Correlation	−0.156	−0.251 **	−0.082	1	−0.175 *	0.008	0.224 *	−0.182	−0.077
	Sig. (1-tailed)	0.071	0.008	0.220		0.049	0.471	0.017	0.084	0.234
	*N*	90	90	90	90	90	84	90	59	90
ED/Hospital Visits/month	Person Correlation	0.089	0.274 **	0.140	−0.175 *	1	0.218 *	−0.399 **	0.141	0.086
	Sig. (1-tailed)	0.202	0.004	0.093	0.049		0.023	0.000	0.144	0.210
	*N*	90	90	90	90	90	84	90	59	90
Vitamin D Tx	Person Correlation	−0.076	0.096	−0.078	0.008	0.218 *	1	−0.072	0.200	−0.455 **
	Sig. (1-tailed)	0.246	0.193	0.239	0.471	0.023		0.258	0.057	0.000
	*N*	84	84	84	84	84	90	84	64	90
Hospitalization rates (survey)	Person Correlation	0.101	0.028	0.095	0.224 *	−0.399 *	−0.072	1	−0.058	−0.201 *
	Sig. (1-tailed)	0.172	0.396	0.186	0.017	0.000	0.258		0.332	0.029
	*N*	90	90	90	90	90	84	90	59	90
Vitamin D Dose Frequency	Person Correlation	0.080	0.055	−0.009	−0.182	0.141	0.200	−0.058	1	0.031
	Sig. (1-tailed)	0.274	0.340	0.472	0.084	0.144	0.057	0.332		0.402
	*N*	59	59	59	59	59	64	59	64	64
Vitamin D Dose Per Week	Person Correlation	−0.044	0.073	−0.025	−0.077	0.086	−0.455 **	−0.201 *	0.031	1
	Sig. (1-tailed)	0.339	0.248	0.408	0.234	0.210	0.000	0.029	0.402	
	*N*	90	90	90	90	90	90	90	64	100

Sickle Cell Disease survey bivariate analysis survey data. Significance is determined by one-way ANOVA correlation. ** Correlation is significant at the 0.01 level (1-tailed) * Correlation is significant at the 0.05 level (1-tailed).

**Table 2:** Chi-squared analysis of vitamin D status compared to vitamin D supplementation.

**A**					
**Vitamin D levels as compared to Vitamin D status**					
VitD Treatment			**Vitamin D Status**		**Total**
			**Insufficient**	**Sufficient**	
	Yes	Count	52	15	67
		Expected count	53.6	13.4	67.0
		% within VitD Treatment	77.6%	22.4%	100.0%
	No	Count	20	3	23
		Expected count	18.4	4.6	23.0
		% within VitD Treatment	87.0%	13.0%	100.0%
Total		Count	72	18	90
		Expected count	72.0	18.0	90.0
		% within VitD Treatment	80.0%	20.0%	100.0%
**B**					
**Chi-Square Tests**					
	**Value**	**df**	**Asymptotic Significance** **(2-sided)**	**Exact Sig.** **(2-sided)**	**Exact Sig.** **(1-sided)**
Pearson Chi-Square	0.934 ^a^	1	0.334		
Continuity Correction ^b^	0.442	1	0.506		
Likelihood Ratio	1.003	1	0.317		
Fisher’s Exact Test				0.546	0.260
Linear-by-Linear Association	0.924	1	0.336		
*N* of alid Cases	90				

There was no observed statistical association of vitamin D status of sufficient (>20 ng/mL), insufficient (<20 ng/mL) and Vitamin D treatment. (**B**) Pearson chi-squared >0.05; ^a^: 1 cells (25%) have expected count less than 5. The minimum expected count is 4.60; ^b^: Computed only for a 2 × 2 table.
